# Is the Association Between Sports Participation in Childhood and Adolescence and Cardiometabolic Risk Mediated by Cardiorespiratory Fitness in Adulthood?

**DOI:** 10.1002/ajhb.70147

**Published:** 2025-09-16

**Authors:** Mariana Biagi Batista, Mileny Caroline Menezes de Freitas, Cynthia Correa Lopes Barbosa, Gabriela Blasquez Shigaki, Catiana Leila Possamai Romanzini, Danielle Venturini, Alessandra Miyuki Okino, Décio Sabbatini Barbosa, Enio Ricardo Vaz Ronque

**Affiliations:** ^1^ School of Education, Federal University of Mato Grosso Do Sul (UFMS), Campo Grande Mato Grosso Brazil; ^2^ Laboratory of Physical Activity and Health, Center of Physical Education and Sports, Londrina State University (UEL) Londrina Brazil; ^3^ Academic Department of Humanities Federal University of Technology Paraná (UTFPR) Apucarana Brazil; ^4^ Department of Physical Education University Paulista (UNIP) São Paulo Brazil; ^5^ Department of Physical Education University Center of Rio Preto (UNIRP) São Paulo Brazil; ^6^ Clinical Analysis Laboratory, Center of Health Sciences, University of Londrina Londrina Brazil

**Keywords:** health, physical activity, physical fitness, sports, young adult, youth

## Abstract

**Objective:**

Analyze the direct and indirect associations between sports participation (SP) during childhood and adolescence and the metabolic risk profile in adulthood. Additionally, investigate whether the relationship is mediated by current levels of cardiorespiratory fitness (CRF).

**Methods:**

A retrospective observational study was conducted with 123 young adults (61 males), aged 18–25 years. Metabolic variables included body mass index (BMI), waist circumference (WC), relative body fat percentage (%BF), systolic blood pressure (SBP), diastolic blood pressure (DBP), and blood markers such as glucose levels, lipid profile, C‐reactive protein, insulin, and the homeostatic model assessment of insulin resistance (HOMA‐IR). A composite metabolic risk score was created by summing the *z*‐scores. CRF was estimated using the 20‐m shuttle run test. SP was assessed using a retrospective questionnaire, while moderate‐to‐vigorous physical activity (MVPA) in adulthood was measured using accelerometry. Structural equation modeling was applied to examine both direct and indirect associations.

**Results:**

SP during childhood, adolescence, and both periods was indirectly and inversely associated with the metabolic risk score and HOMA‐IR in adulthood. The effect of youth SP on metabolic risk was mediated by adult VO_2_ max related to metabolic score (*β* = −0.127; *p* < 0.001) and also to HOMA‐IR (*β* = −0.067; *p* < 0.001). Moreover, MVPA positively interacted with VO_2_ max across all analytical models (*p* < 0.05).

**Conclusion:**

Youth SP during childhood and adolescence was indirectly associated with reduced metabolic risk in adulthood, with this relationship being mediated by current CRF. Additionally, the current practice of MVPA contributes positively to CRF in adulthood.

## 1 Introduction

Non‐communicable diseases (NCDs) are among the leading causes of premature mortality in adults worldwide, representing a significant public health challenge today (Institute for Health Metrics and Evaluation 2024). Research has identified that individual risk factors are directly linked to these morbidity and mortality events. Among them, metabolic risks such as hypertension, diabetes, overweight, and obesity, as well as behavioral risks such as smoking (Institute for Health Metrics and Evaluation 2024) and physical inactivity (Biswas et al. 2022), are particularly relevant. The global prevalence of insufficient levels of physical activity is estimated at 31% (Strain et al. 2024), and over the past three decades, the global burden of diseases attributable to physical inactivity has increased, with approximately 0.83 million deaths linked to this behavior (Xu et al. 2022).

Thus, reducing physical inactivity rates may serve as a strategic approach to lowering risk factors for NCDs and consequently reducing public health costs (Prodel et al. 2023; Santos et al. 2023). Strategies aimed at increasing levels of physical activity (PA) in the population are a key focus of major international organizations and health researchers (WHO 2018), particularly among young populations, as PA is a behavior that tends to exhibit moderate stability throughout life (Hayes et al. 2019).

Although various forms of PA demonstrate potential health benefits, sports participation (SP) has gained increasing attention. In this context, evidence suggests that SP during childhood and adolescence is associated with health risk factors both in early life (Hébert et al. 2017; Telford et al. 2016; Basterfield et al. 2015) and in adulthood (Lima et al. 2014, 2016; Werneck et al. 2019; Silva et al. 2023).

Thus, in addition to confirming the associations between youth SP and health risk factors throughout life in different populations and contexts, it is crucial to clarify how these relationships are actually established. Several theoretical models have been proposed for this purpose, highlighting that PA may exert both direct and indirect positive effects on adult health (Malina 2001; Twisk et al. 2002a; Hallal et al. 2006). Research has demonstrated an indirect effect of physical exercise on mortality, mediated by improvements in cardiovascular risk factors (Bonekamp et al. 2023). Similarly, the relationship between moderate‐to‐vigorous physical activity (MVPA) and lung function is mediated by cardiorespiratory fitness (CRF) (Ostolin et al. 2022).

In this context, research highlights the crucial role of physical fitness in these relationships (Twisk et al. 2002b), particularly CRF, as a potential mediating variable. CRF appears to be more strongly associated with long‐term health outcomes compared to habitual physical activity (Leek et al. 2011). Furthermore, maintaining SP from childhood through adolescence leads to engagement in MVPA in early adulthood (Kwon et al. 2021). Additionally, youth SP is inversely associated with hypertension in adulthood, regardless of MVPA (Silva et al. 2023), and has been linked to reduced body fat and lower C‐reactive protein levels in adulthood (Werneck et al. 2019).

Thus, it is essential to better understand how the traditional model of association between SP in childhood and adolescence and metabolic risk factors in adulthood is established, as well as to test potential alternative analytical models. The aim of the present study was to examine the direct and indirect associations between sports participation in childhood and adolescence and the metabolic risk profile in adulthood, and to test whether this relationship is mediated by CRF or current MVPA.

## 2 Methods

### 2.1 Sample and Study Design

The present study included 123 young adults (61 males) aged 18–25 years. These participants were part of a mixed longitudinal project conducted between 2002 and 2006 in the municipality of Londrina, state of Paraná. After a period of approximately 15 years, they were invited to participate in this research, as previously described by Batista et al. 2024. Sample size was estimated based on the application of linear regression models. The 104 + *n* equation of predictors was used to test them individually (Stevens 1996). The predictors included: SP in childhood, SP in adolescence, persistence in SP during childhood and adolescence, and sex, suggesting an approximate sample of 110 participants of both sexes.

**FIGURE 1:**
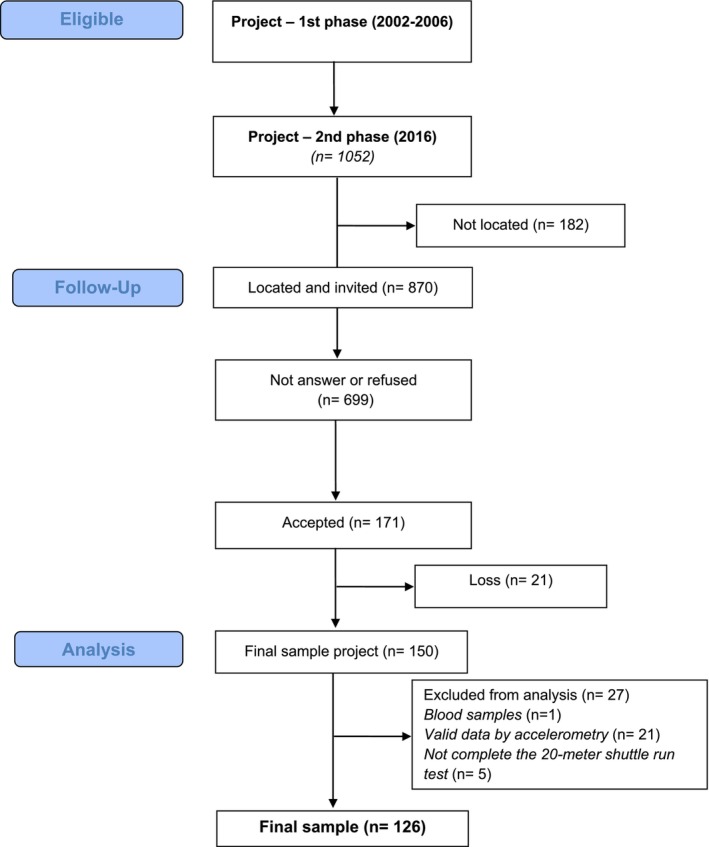
Sample selection consort flow diagram (adapted from Batista et al. 2024).

The inclusion criterion was participation in at least 1 year of the first phase of the project. Exclusion criteria included failure to provide blood samples for determining metabolic risk variables; missing valid data on habitual physical activity in adulthood assessed by accelerometry and failure to complete the 20‐m shuttle run test for estimating oxygen consumption (VO_2_) due to possible physical or health restrictions (five participants were excluded for this reason), described in Figure 1.

Participants were informed about the aims of the study and the procedures to which they would be submitted, and they subsequently signed the Informed Consent Form. The research was approved by the Research Ethics Committee of the State University of Londrina, in accordance with the guidelines of Resolution 466/2012 of the National Health Council on research involving human beings, under Opinion No. 1.340.735 issued on 11/27/2015.

### 2.2 Anthropometry

Body mass was measured using a Balmak digital platform scale, with a precision of 0.5 kg, and height was measured using a Harpenden portable stadiometer, with a precision of 0.1 cm, according to procedures described by Gordon et al. 1988. Body mass index (BMI) was calculated as the body mass/height^2^ ratio (kg/m^2^). Additionally, waist circumference (WC) was measured using a Cardiomed flexible tape measure, with a precision of 1 mm (WHO 2011).

### 2.3 Body Fat

Body fat was assessed using dual‐energy X‐ray absorptiometry (DXA). Relative body fat percentage (%BF) was estimated through whole‐body scanning on a Lunar device, model G.E. PRODIGY—LNR 41.990. All participants were dressed in light clothing, barefoot, and without any metal objects. Participants were positioned in a supine position, aligned, remaining still for approximately 15–20 min.

The equipment calibration followed the manufacturer's recommendations, and both calibration and scan were performed by a technician with experience in this type of assessment. Participants signed the consent form that included information on contraindications and a description of procedures adopted during the examination.

### 2.4 Cardiorespiratory Fitness

As a CRF indicator, participants performed the 20‐m shuttle run test (Léger and Gadoury 1989). Participants were instructed not to use medications, smoke, eat, or consume alcoholic beverages within 2 h prior to the test, and also to refrain from any physical exercise during the 24 h preceding the test. To estimate peak oxygen consumption (VO_2_) in milliliters of oxygen consumed per kilogram of body mass per minute (ml/kg/min), the equation proposed by Léger and Gadoury 1989, was used: VO_2_ peak = −24.4 + 6.0 × (speed in km/h achieved during the test).

### 2.5 Blood Pressure

For the systolic blood pressure (SBP) and diastolic blood pressure (DBP) measurement, the recommendations of the 7th Brazilian Hypertension Guidelines (Sociedade Brasileira de Cardiologia 2016) were followed. To assess resting blood pressure, the OMRON digital device, model HEM‐742, validated for adults (Coleman et al. 2005), was used. Participants were previously instructed to refrain from engaging in any vigorous physical activity, consuming alcoholic or caffeinated beverages in the 24 h prior to the day of measurement, and smoking at least 60 min before the evaluation.

On the day of data collection, prior to measurements, participants were instructed to empty their bladders and remain seated in a chair, resting for 10 min. Three blood pressure measurements were taken with a five‐minute interval between them, using a cuff placed on the right arm in a seated position, with legs uncrossed, feet flat on the ground, and back resting against the chair. The arm was positioned at heart level (midsternal point or 4th intercostal space), with the palm facing upward. The average value of measurements was recorded as the reference.

### 2.6 Blood Variables

Blood collection was performed in an appropriate room, and the analyses were conducted in specialized laboratories at the University Hospital of Londrina, using a vacuum system. Tubes without anticoagulant (for serum collection) with separator gel and tubes containing sodium fluoride for glucose measurement were collected. Blood samples were collected after 12 h of fasting. Laboratory analysis was conducted on the same date as blood collection. It is important to note that the biological material collected for the project was solely and exclusively used for the analyses outlined in this study. This material was not used for genetic studies, and after the completion of the study, disposal was carried out in accordance with the institution's waste management regulations.

Glucose levels, lipid profile (total cholesterol, high density lipoprotein (HDL) cholesterol, the Friedewald equation was used to determine low density lipoprotein [LDL] (LDL = total cholesterol—HDL + triglycerides/5). Very low density lipoprotein (VLDL) was calculated by dividing the triglycerides [TG] value by 5 (VLDL = TG/5), and TG), and C‐reactive protein were measured using a biochemical autoanalyzer (Dimension‐Siemens) and Siemens kits. Insulin levels were determined using enzyme immunoassay with microparticles on the AXSYN (ABBOTT) equipment. The Homeostatic Model Assessment‐Insulin Resistance (HOMA‐IR) was calculated as follows: HOMA‐IR = fasting glucose (mmol/L) × fasting insulin (IU/mL)/22.5 (Meng et al. 2013).

### 2.7 Metabolic Risk Profile in Adulthood

The variables considered for the analysis of the metabolic risk profile, in isolation, were: BMI, WC, %BF, SBP, DBP, VO_2_, glucose, total cholesterol, HDL cholesterol, LDL cholesterol, triglycerides, insulin, C‐reactive protein, and the insulin resistance indicator HOMA‐IR. Additionally, a unique metabolic risk score for the sample (adult metabolic risk score) was determined. For this calculation, the *z*‐score (individual value—group mean/group standard deviation) was initially computed for each of the included variables. The total metabolic risk score represented the sum of the *z*‐scores for the following variables: triglycerides, HDL cholesterol (−1), the mean of SBP and DBP, WC, and HOMA‐IR.

### 2.8 Participation in Sports During Childhood and Adolescence

Information regarding SP during childhood and adolescence indicator was obtained using a retrospective instrument (Fernandes and Zanesco 2010). The childhood period was defined as the age group between seven and 10 years, and adolescence was defined as the age group between 11 and 17 years. Two questions were asked: (1) “Outside of school, did you participate in any organized and supervised sports activities for at least 1 year when you were between 7 and 10 years old?” and (2) “Outside of school, did you participate in any organized and supervised sports activities for at least 1 year when you were between 11 and 17 years old?”

As a result of these questions, the variable was separately analyzed for each phase as follows: SP in Childhood: (a) Yes and (b) No, and SP in Adolescence: (a) Yes and (b) No. Additionally, the responses could reflect SP throughout the entire youth phase, which was dichotomized to generate the variable “Persistence in SP during Childhood and Adolescence”: (a) Both: if the participant answered “yes” to both questions, and (b) No: if the participant answered “no” to any or both questions.

### 2.9 Habitual Physical Activity in Adulthood

The habitual PA of participants was objectively assessed using accelerometry. The accelerometer used was from ActiGraph (ActiGraph, Pensacola, FL), model wGT3X‐BT, and was placed on the right side of the body, near the iliac crest, fixed with an adjustable elastic strap. The device was used for a period of seven consecutive days, removed only during sleep, bathing, and aquatic activities. For the initialization of the accelerometer, the frequency of 30 Hz was selected to record the information. The ActiLife software version 6.13.3 was used for data processing.

Data were considered valid when participants used the accelerometer for at least 480 min per day and for at least 4 full days, including at least one valid weekend day. Non‐use periods were defined as intervals of at least 60 consecutive minutes of zero counts, with a tolerance of one to 2 min of counts between 1 and 100 (Troiano et al. 2008).

Cutoff points used to determine different PA intensities were based on the vector magnitude of the accelerometer, referring to: moderate PA: 2690 to 6166 counts per minute, and vigorous PA: 6167 to 9642 counts per minute (Sasaki et al. 2011). Data were reintegrated by counts per 60 s. To identify the prevalence of adults meeting the recommended levels of MVPA, the recently proposed recommendation was used (Bull et al. 2020). For the purposes of our analyses, participants achieving at least 150–300 min of moderate intensity PA per week, or an equivalent combination of moderate‐ and vigorous‐intensity activity were considered.

### 2.10 Statistical Analysis

The Kolmogorov–Smirnov test was used to assess data distribution. Descriptive statistics, including mean and standard deviation, were used to characterize the sample, with independent Student's *t*‐test applied for sex comparisons. For the description of prevalence variables, frequency percentages were used, and chi‐squared tests were applied for sex comparisons.

Generalized Estimating Equations (GEE) were used to compare descriptive variables and metabolic profiles according to participation in sports during childhood and adolescence. For the GEE analysis, the Gamma distribution was assumed for variables, and all comparisons were controlled by sex.

Structural Equation Modeling was applied to estimate associations (pathways) between SP in childhood, adolescence, and both periods, and the cardiometabolic risk score in adulthood (direct pathways), as well as indirect pathways incorporating latent variables (adult MVPA and VO_2_) as potential mediators. The method of maximum likelihood estimation was applied. Results are presented as regression coefficients (β) and correlation coefficients (r). The Chi‐squared test (*X* ^2^) was used to evaluate the goodness‐of‐fit of models, taking into account the sample size. Data were processed using SPSS, and for Structural Equation Modeling, the AMOS computational package was used. A significance level of *p* < 0.05 was considered for all analyses.

## 3 Results

The descriptive sample characteristics are presented in Table 1. Males had higher body mass, height, and BMI values compared to females. The prevalence of SP in childhood was higher in males than in females (90% vs. 73%; *p* = 0.012), and this difference was not observed for sports participation in adolescence. Regarding the MVPA recommendation in adulthood, more than 60% of the sample did not meet the minimum recommendation of 150–300 min of moderate‐intensity PA per week, with no significant differences between sexes.

**TABLE 1:** Descriptive characteristics of the sample by gender (*n* = 123): continuous variables presented as mean ± standard deviation and categorical variables as percentages.

Variables	Men (*n* = 61)	Women (*n* = 62)	*p*
Age (years)	22.6 ± 1.7	22.2 ± 1.7	0.315
Body mass (kg)	75.2 ± 10.5	60.1 ± 10.8	< 0.001
Height (cm)	176.3 ± 6.8	164.7 ± 6.8	< 0.001
BMI (kg/m^2^)	24.2 ± 2.9	22.1 ± 3.4	< 0.001
*SP childhood* Yes (%) No (%)	90.2 9.8	72.7 27.4	0.012
*SP adolescence* Yes (%) No (%)	88.5 11.5	75.8 24.2	0.066
*Recommendation MVPA* ^a^ Meet (%) Not meet (%)	36.1 63.9	32.3 67.7	0.656

*Note: p* = Statistical significance regarding the comparison between genders was assessed using independent *t*‐tests for continuous variables and the Chi‐square test for categorical variables.

Abbreviations: BMI = body mass index; MVPA = moderate‐vigorous physical activity; SP = sports participation.

^a^ AT least 150–300 min of moderate‐intensity PA per week.

The comparisons established by GEE between descriptive characteristics and metabolic profile variables, according to participation in sports during childhood and adolescence, are shown in Table 2. In childhood, those who reported participation in sports were younger (*p* = 0.010) and, for the metabolic risk profile, they had lower body fat percentage (*p* = 0.001) and higher values in cardiorespiratory fitness indicators (*p* < 0.001). Additionally, when analyzing sports participation in adolescence, the only statistically significant difference identified was for VO_2_ (*p* < 0.001), with higher values once again observed in the group that reported engagement in sports during this phase of life.

**TABLE 2:** Descriptive characteristics and variables of the metabolic profile, according to participation in sports during childhood and adolescence (*n* = 123).

Variables	Sports participation in childhood			Sports participation in adolescence		
	Yes (*n* = 100)	No (*n* = 23)	*p*	Yes (*n* = 101)	No (*n* = 22)	*p*
Age (years)	22.26 ± 1.65	23.10 ± 1.40	**0.010**	22.45 ± 1.64	22.18 ± 1.63	0.458
BM (kg)	69.29 ± 13.67	65.98 ± 16.74	0.366	69.51 ± 14.26	64.74 ± 13.89	0.137
Height (m)	171.06 ± 8.53	167.98 ± 10.24	0.107	170.74 ± 8.98	169.31 ± 8.72	0.324
BMI (kg/m^2^)	23.38 ± 3.29	23.17 ± 4.47	0.826	23.54 ± 3.54	22.39 ± 3.28	0.133
WC (cm)	75.84 ± 9.56	73.49 ± 9.67	0.281	76.05 ± 9.56	72.36 ± 9.32	0.086
BF (%)	27.37 ± 9.96	34.34 ± 8.81	**0.001**	28.25 ± 9.98	30.43 ± 10.68	0.368
SBP (mm/Hg)	118.80 ± 13.44	113.04 ± 9.39	**0.013**	117.67 ± 12.57	118.27 ± 15.09	0.858
DBP (mm/Hg)	68.69 ± 8.14	70.71 ± 6.35	0.184	68.78 ± 8.14	70.41 ± 6.37	0.289
VO_2_ (ml/kg/min)	40.94 ± 7.14	35.21 ± 6.41	**< 0.001**	40.83 ± 7.31	35.46 ± 5.82	**< 0.001**
Glucose (mg/dL)	88.39 ± 5.88	87.30 ± 4.59	0.322	88.29 ± 5.78	87.73 ± 5.22	0.644
Total‐C (mg/dL)	177.23 ± 33.63	177.87 ± 34.76	0.935	177.57 ± 31.78	176.23 ± 42.60	0.886
HDL‐C (mg/dL)	55.93 ± 15.24	60.17 ± 16.53	0.249	57.02 ± 15.32	55.14 ± 16.62	0.617
LDL‐C (mg/dL)	105.57 ± 29.84	100.23 ± 27.46	0.396	104.40 ± 27.15	105.64 ± 39.21	0.885
TRIG (mg/dL)	78.60 ± 42.48	87.35 ± 33.01	0.268	80.78 ± 41.04	77.23 ± 41.40	0.708
Insulina (Ui/mL)	7.85 ± 3.53	7.63 ± 3.38	0.772	7.77 ± 3.35	7.98 ± 4.23	0.829
C‐RP (mg/L)	2.16 ± 2.74	2.71 ± 1.79	0.217	2.23 ± 2.73	2.41 ± 1.84	0.698
HOMA‐IR	1.73 ± 0.83	1.65 ± 0.74	0.630	1.71 ± 0.78	1.73 ± 0.94	0.913

*Note:* Values presented as mean and standard deviation. All comparisons by GEE were controlled for sex. Values in bold indicate *p* < 0.05.

Abbreviations: BF = body fat using the dual‐energy radiological absorptiometry technique; BM = body mass; BMI = body mass index; C = cholesterol; HDL = high density lipoprotein; HOMA‐IR = homeostatic model assessment for insulin resistance; LDL = low density lipoprotein; PCR = C‐reactive protein; TRIG = triglycerides; VO_2_ = oxygen consumption estimated by the 20‐m shuttle run test; WC = waist circumference.

**FIGURE 2:**
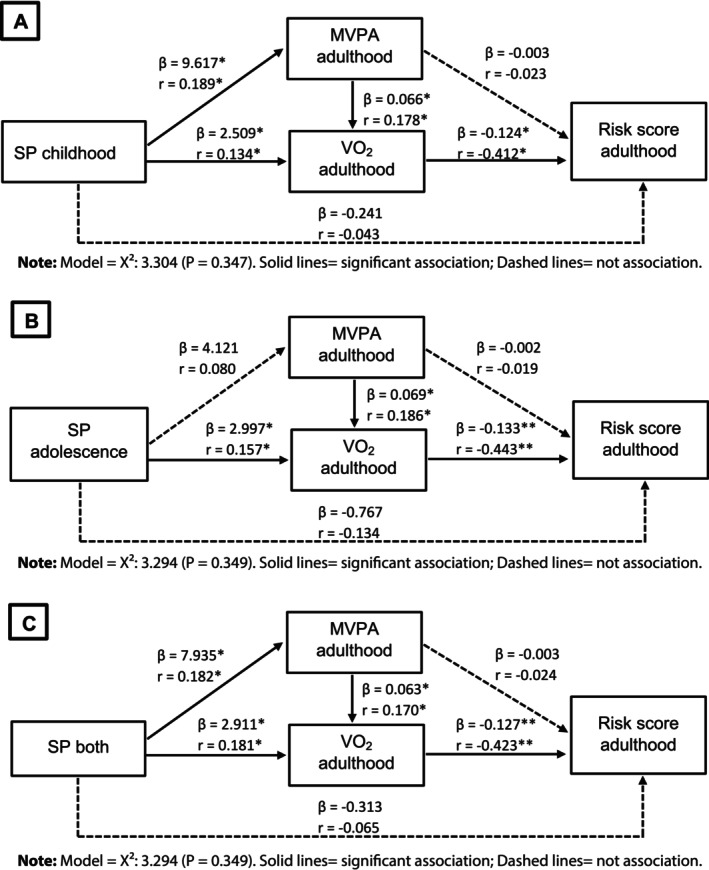
Association models examining the relationship between sports participation during childhood, adolescence, or both, and the metabolic risk score in adulthood (*n* = 123).

**FIGURE 3:**
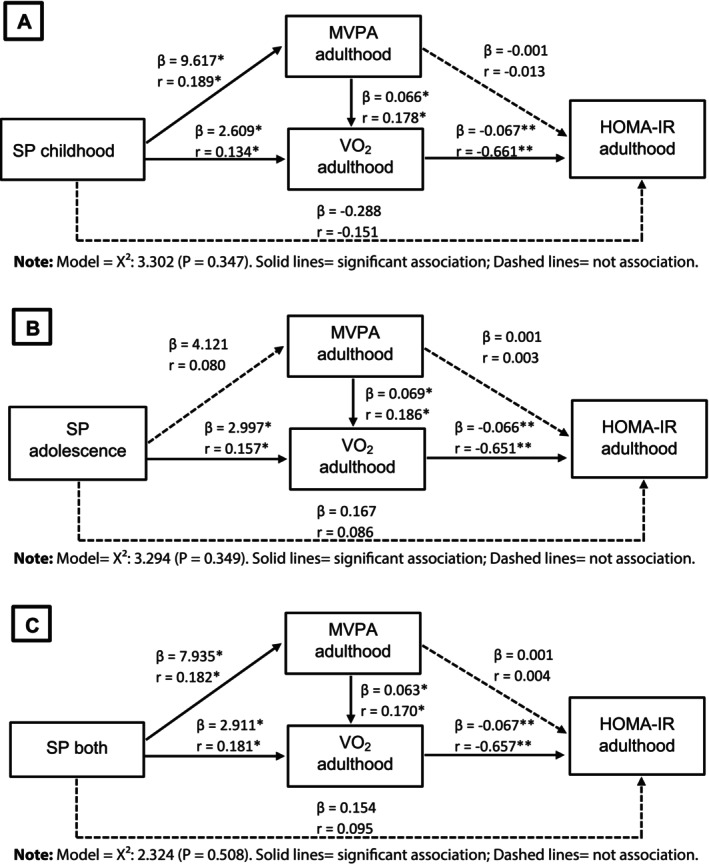
Association models examining the relationship between sports participation during childhood, adolescence, or both, and the insulin resistance index (HOMA‐IR) in adulthood (*n* = 123).

The results of the association analysis using structural equation models between SP during childhood, adolescence, and both phases and metabolic risk in adulthood are presented in Figures 2 and 3. Solid lines represent significant associations, while dashed lines indicate the absence of association. The models tested between SP during childhood (*β* = −0.124) [Figure 2A], adolescence (*β* = −0.133) [Figure 2B], and both phases (*β* = −0.127) [Figure 2C] and the metabolic risk score suggest inverse associations, mediated by adult VO_2_. This indirect pathway accounted for 17%, 20%, and 18%, respectively, of the total variation in the relationship between SP in childhood and the cardiometabolic risk score. Furthermore, regardless of SP during childhood and adolescence, MVPA in adulthood is positively associated with VO_2_, which in turn reduces metabolic risk in this phase of life.

The same pattern was observed for the HOMA‐IR index, where the relationship between SP during childhood (*β* = −0.067) [Figure 3A], adolescence (*β* = −0.066) [Figure 3B], and both phases (*β* = −0.067) [Figure 3C] showed an indirect association, mediated by VO_2_ which was inversely associated with HOMA‐IR in adulthood. This indirect pathway explained between 42% and 44% of the variance. Additionally, MVPA in adulthood was positively related to current VO_2_.

## 4 Discussion

The main results of the present study demonstrated that SP during childhood, adolescence, and both phases was indirectly associated with metabolic risk profiles in adulthood. SP in childhood and adolescence was partially mediated by VO_2_ in adulthood, which in turn interacted with MVPA in adulthood in all analysis models. In addition, VO_2_ was directly associated with both the metabolic risk score and HOMA‐IR in adulthood. It is important to note that SP during childhood resulted in better body fat and VO_2_ indicators in adulthood, while SP in adolescence showed higher VO_2_ values, regardless of sex.

Based on theoretical models that highlight the role of adult PA and physical fitness in adults in the relationship between PA in youth and health status in adulthood (Malina 2001; Twisk et al. 2002a; Hallal et al. 2006), this study aimed to verify the hypothesis of a possible indirect relationship—mediated by adult variables (MVPA and VO_2_)—as well as a direct relationship between PA in youth and metabolic risk profile in adulthood. The main findings support the hypothesis of an indirect relationship, indicating that SP in childhood and adolescence was indirectly associated with metabolic outcomes in adulthood, as VO_2_ mediated these relationships, and that current MVPA contributes positively to VO_2_ in adulthood.

The direct association between PA in childhood and health in adulthood is low to moderate (Malina 2001, 2025; Twisk et al. 2002b), which partially corroborates our results, in which the direct relationship between PA and metabolic risk was not significant. Malina (2025) highlights that maintaining CRF in adulthood emerges as one of the main mediators of health benefits derived from early practice of PA, as well as the need to consider other behaviors that influence both physical fitness and health status in adults, such as regular PA. These facts were observed in the present study, since VO_2_ in adulthood indicated an inverse association with metabolic risk, and MVPA positively contributed to VO_2_ in adulthood.

The role of PA in adult health was highlighted in a pioneering study conducted by Paffenbarger et al. (1986), which demonstrated that increased PA intensity reduces all‐cause mortality in different age groups. In this sense, the practice of SP during childhood and adolescence, even if not directly related to health risk factors in adulthood, has proven to be an important strategy for increasing VO_2_ levels (Haynes et al. 2021) and PA in adulthood, particularly at moderate to vigorous intensities (Batista et al. 2024). This result reinforces that, throughout life, PA is essential to maximize health benefits, especially those related to cardiovascular risk factors in adulthood (Camhi 2013). In addition, promoting and maintaining PA in young people can improve CRF in adulthood, which are key elements for health promotion (Malina 2025).

Therefore, encouraging the engagement of young individuals in sports should be reinforced, as it may ensure greater participation in moderate‐to‐vigorous intensity activities compared to those who are not engaged in sports (Koorts et al. 2019). Additionally, evidence suggests that higher‐intensity PA serves as a protective factor in preventing cardiovascular events (Lin et al. 2023) and contributes to better health risk indicators in adulthood, including reduced waist circumference, improved glucose regulation, and lower levels of inflammatory cytokines (Liu et al. 2022), as well as a lower risk of mortality (Lee et al. 2022; Tarp et al. 2024).

Thus, based on the findings of the present study, theoretical reasoning can be developed to explain the indirect relationships specifically observed for SP. Initially, SP during childhood and adolescence may be associated with higher levels of MVPA and improved CRF indicators even at this stage of life (Telford et al. 2016). For children and adolescents, a dose–response relationship has been established between levels of MVPA and various health benefits, forming the basis of current international recommendations (Bull et al. 2020). Consequently, significant health benefits can already be achieved during childhood and adolescence, aligning with the first pathway suggested in traditional association models (youth SP/youth health).

Following the previously mentioned theoretical framework, it is evident that PA‐related behaviors adopted early in life—including SP as one of its key domains—can be reasonably maintained into adulthood (Hayes et al. 2019). This relationship was identified in the present study through the associations observed between youth SP and adult MVPA, further corroborated by several previously published studies (Mäkelä et al. 2017; Tammelin et al. 2003; Telama 2009). This finding illustrates the second pathway proposed in indirect association models (youth SP/adult PA).

Finally, in the present study, both youth SP and adult MVPA were associated with VO_2_ in adulthood, a finding also supported in the literature, though more consistently in adulthood (Dencker et al. 2006; Leskinen et al. 2009). Compared to other physical fitness components and PA itself, CRF has been more strongly linked to health risk factors, morbidity, and mortality due to cardiovascular diseases and all‐cause mortality (Ross et al. 2024; Kokkinos et al. 2022). This may, in part, explain the significant mediating role of VO_2_ in the relationship models tested in the present study.

All these favorable conditions involving MVPA and VO_2_ in adulthood may have contributed to maintaining lower body fat levels among individuals who reported engagement in SP, as identified in the comparisons performed in this study. Consequently, this study contributed to the identification of a better metabolic risk score and HOMA in the group of adults who participated in sports during childhood and adolescence (Fernandes et al. 2015). Therefore, these findings support the conclusion of the theoretical rationale, confirming the hypothesis of an indirect relationship established through the second pathway within indirect association models:

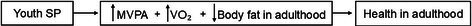

*Source:* Created by the author.

The present study aims to clarify how associations of a specific type of PA, represented by SP during childhood and adolescence, are established with health risk factors in adulthood. This is based on hypotheses suggested by classical models of relationships in literature, considering two stages of life. The objective assessment of PA in adulthood is also considered a mediating variable in this association, ensuring more reliable results when compared to subjective methods. However, limitations should be considered, such as the absence of data on habitual physical activity during childhood and adolescence, which could shed light on the possibility that indirect associations may occur, suggesting the impact of SP on the maintenance of the levels of PA from childhood and adolescence to adulthood. Despite the retrospective assessment of SP, sports offer advantages related to recall when compared to other activities.

## 5 Conclusion

It was concluded that SP in childhood and adolescence was indirectly and inversely associated with metabolic risks in adulthood, with this relationship being mediated by current CRF. Furthermore, current MVPA is an important factor that is associated with higher CRF levels in adulthood.

## Author Contributions

**Mariana Biagi Batista**, **Mileny Caroline Menezes de Freitas:** conception, desing, acquisition of data, analysis and interpretation. **Cynthia Correa Lopes Barbosa**, **Gabriela Blasquez Shigaki**, **Catiana Leila Possamai Romanzini:** acquisition of data, drafting and review. **Danielle Venturini**, **Alessandra Miyuki Okino**, **Décio Sabbatini Barbosa:** blood analysis and review. **Enio Ricardo Vaz Ronque:** review, supervision, editing, is the guarantor of the work and, as such, had full access to all the data of the study, assuming full responsibility. All authors reviewed the manuscript final.

## Conflicts of Interest

The authors declare no conflicts of interest.

## Data Availability

The data that support the findings of this study are available from the corresponding author upon reasonable request.
